# Trans-arterial hepatic radioembolisation of yttrium-90 microspheres

**DOI:** 10.2349/biij.2.3.e43

**Published:** 2006-07-01

**Authors:** R Murthy, A Habbu, R Salem

**Affiliations:** 1 Interventional Radiology Section, University of Texas, Houston, United States; 2 Department of Radiology, Northwestern Memorial Hospital, Chicago, United States

**Keywords:** Liver cancer, yttrium-90, radioembolisation, colon cancer, hepatocellular cancer

## Abstract

The liver represents a frequent site for metastatic disease, in addition to being a site for primary cancer. Hepatic metastases from certain neoplasms, such as colon, neuroendocrine, melanoma and gastrointestinal stromal tumour have a distinct predilection to metastasize the liver, which in many cases may represent the only or the dominant site of disease. In these circumstances, cytoreduction via surgery or *in situ* ablative techniques aims to influence the natural history of the disease progression and improve clinical outcomes.

Liver directed therapy utilising yttrium-90 microspheres represents a recently introduced *in situ* multidisciplinary cancer therapy that has caught the attention of many physicians faced with the challenges of treating these complex patients. Although similar to other forms of trans-arterial liver directed therapy, there are discrete differences and potentially fatal treatment consequences unique to this therapy. This objective of this review article is to provide the reader a basis for understanding the therapeutic principles, patient exclusion criteria, pre and post therapy investigations and salient clinical results in the two most commonly treated disease types; metastatic colorectal cancer and hepatocellular cancer.

## INTRODUCTION

Metastatic colorectal cancer and hepatocellular cancer are amongst the commonest causes of cancer mortality worldwide. A total of 437,000 worldwide deaths are estimated for colorectal cancer, making it the third most important cause of cancer mortality overall [1]. Hepatocellular cancer is the fourth most common cause of death from cancer and rapidly increasing in incidence in the United States [2]. Surgical resection with curative intent with or without adjuvant chemotherapy for colorectal cancer is considered to offer the highest survival rates that range from 30-58% at five years [3,4]. Recurrent, most often unresectable disease in the hepatic remnant contributes significantly to this inability to achieve long term cure rates for colorectal cancer patients [5]. Five year survival estimates range between 30-50% following hepatic resection and less than 20% following transplantation for hepatocellular cancer [6].

Since mortality and morbidity in this patient group is directly related the presence of hepatic disease, the local application of *in-situ* cytoreductive therapies may favourably alter the natural history of tumour progression. These therapies can be broadly categorised as those applied via the transcapsular or trans-vascular routes. The myriad of therapies that exploit the trans-arterial route are based on the premise that metastatic tumours receive their blood supply from the arterial rather than the portal circulation, unlike normal hepatocytes [7]. Hepatic artery injection allows preferential delivery of material to the peri-tumoural vascular plexus [8]. A suspension of particles injected via the hepatic artery, such as microspheres of appropriate diameter, will preferentially lodge in the peri-tumoural vessels, a process termed embolisation.

Radiation is tumouricidal if sufficient tumour doses can be delivered selectively without damaging adjacent normal tissue in the process. External beam hepatic radiotherapy is limited in efficacy in the presence of multifocal or large tumours in the liver since the radiation exposure of normal hepatocytes results in liver insufficiency before achieving tumour kill [9]. Brachytherapy, wherein the therapeutic radiation source is in physical contact with the tumour, circumvents the limitation of non-selectivity of extracorporeal radiotherapy. The utilisation of this effective technology, however, is largely limited, by the frequent requirement of direct visualisation of the liver that is traditionally achieved intra-operatively and is technically prohibitive in the presence of multifocal disease.

From the above discussion, it is evident that the altered arterial supply to hepatic tumours could potentially be exploited to deliver lethal doses of radiation. A high energy radiation source combined with an appropriately sized trans-hepatic arterial administered embolic microscopic particle would allow radiation to be delivered preferentially to the tumour [10]. A β-emitter, such as yttrium-90, would create a zone of high radiation exposure confined to the vicinity of the tumour while maintaining non-tumourous hepatic parenchymal exposure to tolerable levels. This forms the premise for the selective internal radiation therapy or SIRT. Millions of microspheres, measuring about 30µ in diameter incorporating yttrium-90, are injected via a hepatic arterial catheter to the arterial supply of the tumour. SIRT is a technique that allows high average doses of radiation (200 to 300 Gy) to be given to liver tumours with minimal serious effect on the nontumourous liver [11].

## BIOPHYSICAL PROPERTIES OF YTTRIUM-90

**Figure 1:**
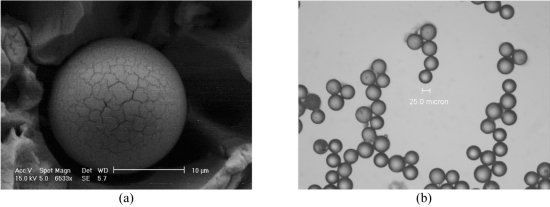
(a) Electron micrograph; SirSpheres, resin. (b) Electron micrograph; Therasphere, glass.

Yttrium-90 (Y90), a pure β emitter is produced by neutron bombardment of yttrium-89 in a reactor. Y90 has a physical half-life of 64.2 hours (2.67 days) and decays to stable zirconium 90. The average energy of the emissions from the Y90 is 0.9367 MeV, with an average/maximal penetration range of 2.5 mm and 11mm respectively in tissue. One gigabecquerel (27 mCi) delivers a total absorbed radiation dose of 50 Gy/kg. In therapeutic use in which the isotope decays to infinity, 94% of the radiation is delivered in 11 days. Y90 is the active moiety in a variety of targeted radio-immunotherapies used in the treatment of a variety of solid organ and hematological malignancies. Two Y90 microsphere products are commercially available (Figure 1), TheraSpheres® (MDS Nordion, Ottawa, Canada) and SIR-Spheres^®^ (SIRTEX Medical, Sydney, Australia) and vary in their physical composition and radioactivity levels (Table 1).

**Table 1:** Characteristics of Microspheres

**Parameter**	**Resin**	**Glass**
Trade name	Sir-Spheres	TheraSphere
Diameter	22 ± 10µm	32 ± 10µm
Specific Gravity	1.6 g/dl	3.6 g/dl
Activity per Particle	50 Bq	2500 Bq
Number of microspheres per averaged administered activity	40 – 80 million	1.2 – 8 million
Material	Resin with bound yttrium	Glass with yttrium in matrix

**Table 2:** Contraindications

**Contraindication**	**Criterion**
Absolute	Exaggerated hepatopulmonary shunting
Absolute	Propensity for gastrointestinal reflux of spheres
Absolute	Pregnancy
Absolute	Concomitant Capecitabine therapy (SIR-Spheres)
Relative	Prior hepatic radiotherapy
Relative	Portal hypertension
Relative	Complete portal vein thrombosis
Relative	Hepatic insufficiency
Relative	Renal insufficiency

## MULTIDISCIPLINARY TREATMENT APPROACH

In order to deliver Y90 microspheres safely and effectively, harnessing the skills of many different specialties are paramount. In the United States, interventional radiologists, surgical oncologists, medical oncologists, nuclear medicine physicians, radiation oncologists, medical physicists and radiation safety experts bring invaluable expertise to the treatment process. It is imperative that this multidisciplinary team confirms the presence of liver dominant unresectable disease before proceeding. In general, a performance status (ECOG ≤ 1) is correlated with reasonable life expectancy.

## CONTRAINDICATIONS

**Figure 2:**
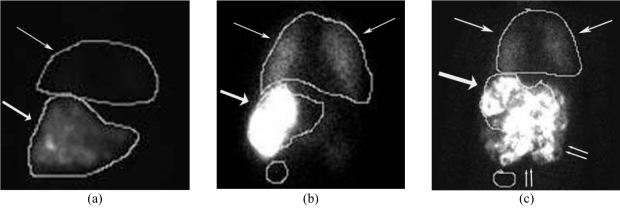
Planar nuclear medicine scintigraphy. Patterns of distribution of Tc99m MAA following hepatic arterial delivery (bold arrow-liver; thin arrow- lung; double arrows- gut). (a) Normal distribution with no extrahepatic activity and no significant hepato-pulmonary shunting. (b) Excessive hepato-pulmonary shunting. A 26% shunt fraction precluded treatment in this patient with hepatocellular carcinoma. (c) Significant gastrointestinal reflux. Treatment is precluded if this phenomenon is not corrected. Incidentally is also noted of excessive hepatopulmonary shunting.

There are two absolute contraindications for liver directed therapy with Y90 microspheres; *excessive hepatopulmonary* and *demonstrable gastrointestinal shunting* that can lead to fatal and morbid complications of radiation pneumonitis and gastric ulceration respectively. Fortunately the likelihood of developing these complications can be detected before definitive treatment by utilising Tc99m MAA as a surrogate that mimics the distribution of the Y90 microspheres (Figure 2).

**Hepatopulmonary Shunting:** Pathologic arteriovenous communications develop within hepatic neoplasia. Microspheres injected into the hepatic artery pass through the tumour via these shunts and are carried by the venous return to the heart via the hepatic veins and eventually embolise within terminal pulmonary arteriolar branches. As the magnitude of shunt increases, proportionately larger numbers of radioactive microspheres can reach the lungs causing clinically significant radiation pneumonitis. Maintenance of lung exposure below a mean dose of 30Gy avoids this complication [12]. The magnitude of this shunting phenomenon is calculated by a quantitative assessment of the ratio of the gamma emission count in the lung to that in the liver corrected for background. This numerical value assists in activity modification when the resin microspheres are used.

**Gastrointestinal Tract Deposition:** Numerous named and unnamed arteries that supply the adjacent gastrointestinal tract arise normally and in a variant fashion from the hepatic arteries. The most common of these arteries are the gastroduodenal and right gastric. During the delivery process, microspheres may inadvertently reflux into these vessels resulting in their embolisation into the gastrointestinal submucosal visceral arterioles. By virtue of the combined ischemia and radiation effect, varying degrees of inflammation and ischemia ensue resulting in an ulcerative diathesis that is frequently treatment refractory. These vessels are therefore prophylactically coil embolised at their origin at the time of the performance of the Tc-99m-MAA shunt study thereby occluding the avenue for microsphere transit to these extrahepatic locations and reducing the incidence of this complication [13].

## PRE- THERAPY INVESTIGATIONS & THERAPY PLANNING

### Lab Analysis

Serum chemical analyses also are performed to evaluate hepatic and renal function, traditionally measured by serum bilirubin and creatinine respectively. The presence and magnitude of elevation of tumour markers specific to the tumour type being treated are ascertained. By consensus, an elevated serum bilirubin level is considered a relative contraindication to treatment with Y90 microspheres. In the presence of renal insufficiency, care must be taken to avoid or minimize the use of iodinated contrast material. Treatment with Y90 microspheres must be based on cross-sectional images and arteriograms in the individual patient. The work-up should include CT or MR imaging of the liver for assessment of tumoural and non-tumoural volume, portal vein patency, and extent of extrahepatic disease.

### Pre-procedural Cross Sectional Imaging with CT or MRI

**Figure 3:**
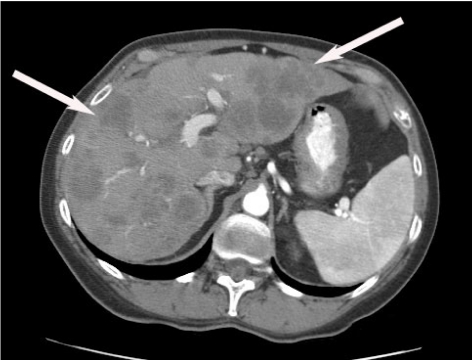
Pre-Y90 microsphere therapy planning evaluation in a 58 year old female with hepatic metastatic colorectal cancer. Contrast enhanced axial CT scan demonstrates numerous bilobar low attenuation lesions consistent with metastases. Note patency of the portal vein.

A triple phase CT to delineate the geographical distribution, the volume and the partition between hepatic parenchyma and tumour is essential in therapy planning (Figure 3). Adjunct information on portal vein patency and aberrant hepatic arterial anatomy is obtained. Distribution of the disease is typically characterised as unilobar or bilobar, however the correlation of tumour with hepatic arterial supply is variable and can only be ascertained with arteriography. Ascites indicates poor hepatic reserve or peritoneal metastasis, both of which have a poor prognosis.

### Arteriographic Assessment and Hepatic Arterial Injection of Tc 99m MAA

**Figure 4:**
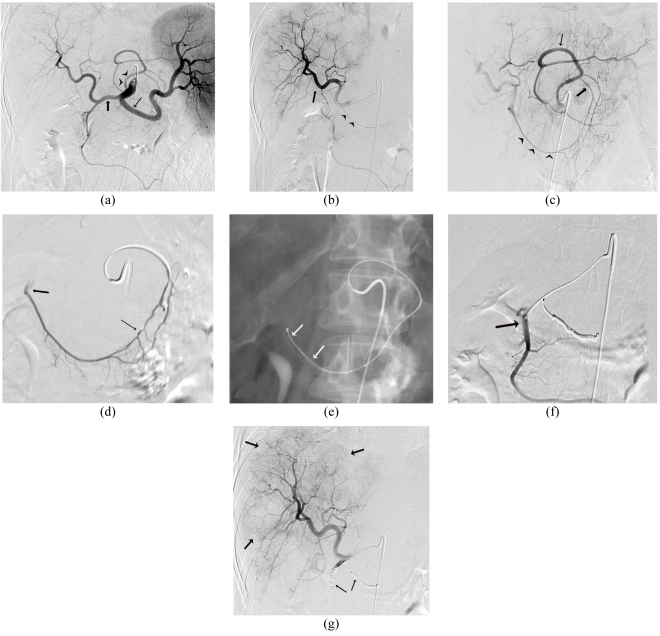
58 year female with hepatic metastatic colorectal cancer undergoing pre-treatment therapy planning arteriography and embolisation of vessels supplying the gastrointestinal tract. (a) Celiac Arteriogram with variant anatomy; showing Splenic artery (thin arrow) ; Common hepatic artery (bold arrow) and the variant Gastrohepatic trunk (arrow heads). (b) Right Hepatic Arteriogram; Catheter tip in right hepatic artery(bold arrow); Right gastric artery (arrow heads) could not be selectively catheterised for embolisation. (c) GastroHepatic Trunk Arteriogram; Left gastric artery (bold arrow)anastomosing with right gastric artery (arrowheads) along the lesser curvature of the stomach ; note left hepatic artery (thin arrow) supplying the left lobe of liver. (d) Left Gastric Arteriogram; Selective 3 Fr microcatheter in the left to right gastric artery arcade(thin arrow); Note reflux into the right hepatic artery at the origin of the vessel (bold arrow). (e) Right Gastric Artery embolisation; two 3 mm fibred platinum microcoils deployed from origin of right gastric artery. (f) Gastroduodenal arteriogram; selective 3 Fr microcatheterisation of origin in preparation for embolisation. (g) Right Hepatic Arteriogram following embolisation of the right gastric artery; Gastroduodenal artery (arrow heads) demonstrating absence of flow to the gastrointestinal tract; Note the diffuse hypervascular hepatic metastases (arrows).

**Figure 5:**
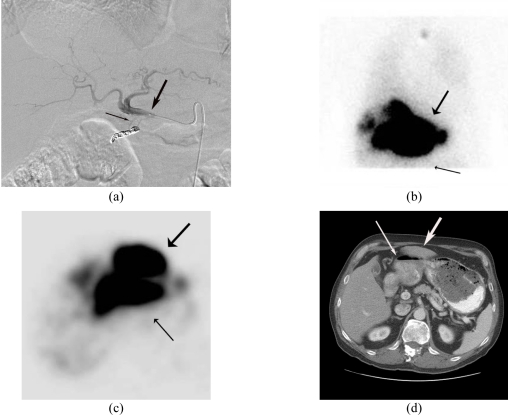
67 year old male with hepatic metastatic colorectal cancer undergoing pre-treatment hepatopulmonary shunt evaluation. (a) Proper Hepatic Arteriogram; Tc99 MAA was injected via a microcatheter placed in the proper hepatic artery (arrow) following coil embolisation of the gastroduodenal artery; Note opacification of the patent right gastric artery (arrow heads). (b) Planar thoracoabdominal scintigraphy following hepatic arterial injection of Tc99 MAA demonstrates predominant left lobe hepatic activity only (bold arrow) and absence of extrahepatic activity (thin arrow). (c) SPECT images (a) demonstrate activity corresponding to the CT (b) images of the left lobe of the liver (bold arrow) and in the stomach (thin arrow) not noted on the planar image.

Arteriography is essential to map the hepatic arterial supply from the celiac and the superior mesenteric artery and is the single most important test to exclude preventable complications. Using a percutaneous inserted catheter, the hepatic arteries are accessed and the supply to the liver and the adjacent gastrointestinal tract is identified. Once identified, these gastrointestinal tract arteries are coil embolised to ensure prevention of reflux of microspheres into the gut (Figure 4). When such arteries are not confirmed arteriographically, the hepatic arterial infusion of 5 mCi of Tc-99m-MAA assists to identify occult extrahepatic perfusion. This is manifested by extra-hepatic scintigraphic activity on nuclear medicine imaging. The culprit artery can usually be retrospectively identified on the angiogram and then embolised before the Y90 microsphere delivery (Figure 5).

## Y90 MICROSPHERE TREATMENT

Solitary or multiple lesions distributed in a lobe or both lobes can be treated with single and multiple microsphere treatments successfully. Nomenclature for the current convention for whole liver treatment by first treating one lobe and then the other in 4-6 weeks is termed “sequential” or “lobar” delivery; as opposed to the whole liver at one setting in which case it is termed ‘bilobar’ in the absence of a lobectomy. The current practice in the United States is to allow a 4-6 week interval between infusions if treatment was intended to be delivered sequentially to allow for recovery after any treatment related toxicities.

## ACTIVITY DETERMINATION

Y90 microspheres are unlike a traditional radiopharmaceutical or brachytherapy device and share the characteristics of both. At present the dose calculation methodology described in the package insert is recommended, however improvements in dosimetry represent an area of intense investigation [14]. CT treatment planning with reconstruction of the liver volumes assists to calculate the required activity for treatment.

**Glass Y90 Microsphere Activity Calculation & Delivery**: The dose determination for glass microspheres is based on a nominal average target dose (150 Gy/kg), and the patient's liver mass is determined from the CT data and assumes the uniform distribution of the microsphere throughout liver volume as:

$$A{\left(GBq\right)}_{glass}=\frac{D(Gy)\times M(kg)}{50}$$

In this equation, *A* is the activity, *D* is the nominal target dose, and *M* is the mass of the targeted liver tissue.

**Resin Y90 Microsphere Activity Calculation and Delivery**: Resin microspheres are received in a vial as a 3 GBq dose, and the individual medical centers remove the prescribed activity. This process differs from that for glass microspheres where a predetermined dose is delivered to the facility. Due to the higher specific activity with glass microspheres and therefore the relative low volume of the spheres per dose, embolic occlusion of the parent artery has not been observed arteriographically. However, the prescribed activity of resin spheres cannot always be delivered completely [15] due to embolic arterial occlusion. In these instances, the residual activity in the delivery vial is measured and the delivered dose is the difference between the prescribed and the residual dose. The manufacturer recommends one of the two methods for activity determination for the resin microsphere; the Body Surface Area method (BSA) and the Empiric Method (EM). However, most experienced practicing physicians recommend the use of the BSA for resin microsphere dose calculation since the delivered dose more closely resembles the activity calculated by the BSA methodology.

### Body Surface Area Method

$$BSA(m^{2})=0.20247\times height{\left(m\right)}^{0.725}\times weight{\left(kg\right)}^{0.425}$$

$$Activity(GBq)=(BSA-0.2)+\frac{VolumeofTumour\times 100}{LiverVolume}$$

### Empiric Method

Activity calculated for whole liver delivery based on tumour replacement as demonstrated on CT.

*Tumour Volume *≤ 25% = 2*GBq*

>25%&≤50% = 2.5*GBq*

>50% = 2.5*GBq*

## BREMSSTRAHLUNG SCAN

**Figure 6:**
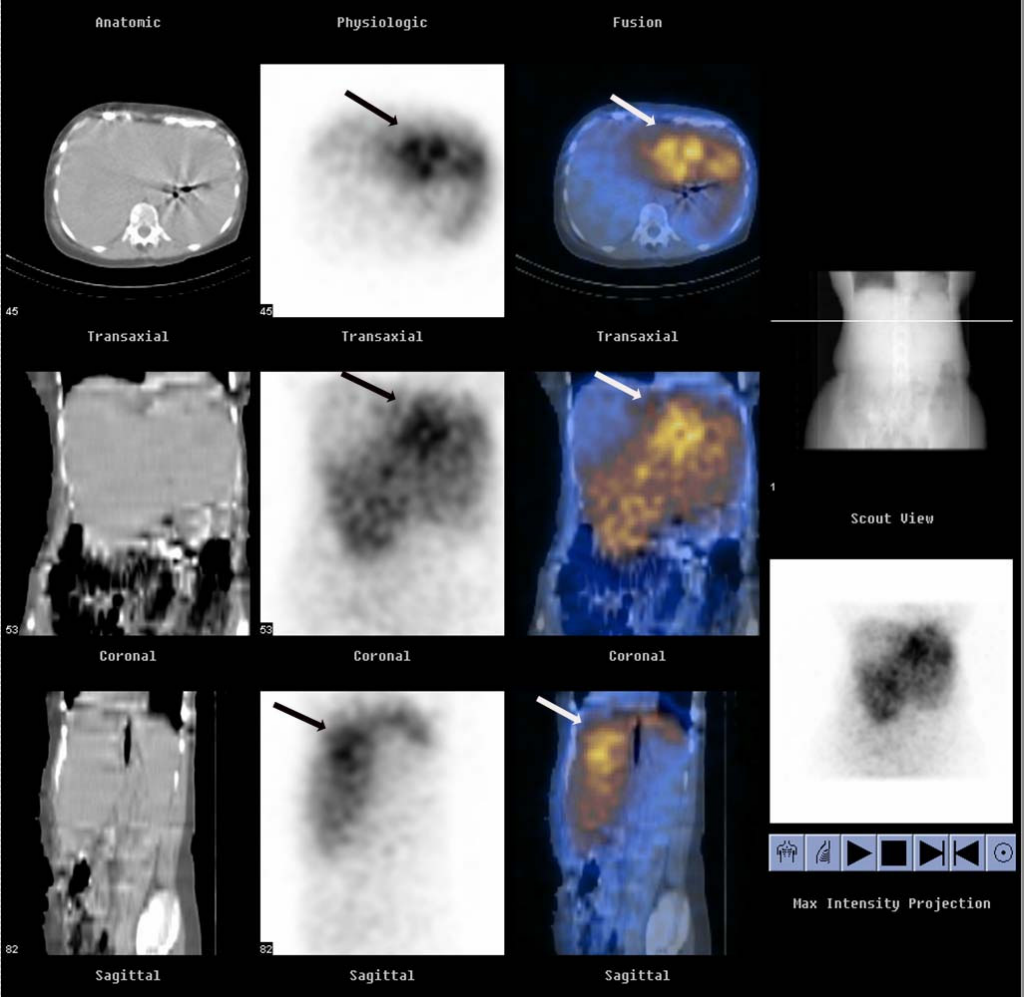
Bremsstrahlung imaging assists in confirming successful targeting of hepatic metastatic deposits from carcinoid tumour. Representative axial, coronal and sagittal CT images(first column), SPECT images (second column) and SPECT/CT fusion images (third column) obtained with a dual-modality imaging system) Hawkeye GE Medical Systems, Milwaukee, Wis) show selective activity in the left hepatic lobe (arrow) approximately 24 hours after intraarterial infusion of 90Y- bearing resin microspheres at a dose of 23.9 mCi (884.3 MBq).

Secondary gamma emission, Bremsstrahlung, scans are possible due to the interaction of the high energy Beta emission interacting with matter. Unfortunately, such Bremsstrahlung emissions represent a broad spectrum of energy emissions rendering relatively poor point to point discrimination. Currently the planar and/or SPECT images obtained from such an acquisition are mostly qualitative and allow the operator to discern the relative distribution of the Y90 microspheres within the liver. Extrahepatic activity may warn clinicians of impending gastrointestinal complications and serve as a quality assurance tool (Figure 6).

## POST TREATMENT COURSE

The most common side effect following treatment is mild to moderate fatigue and abdominal pain generally lasting less than 2 weeks. Nausea and vomiting are less common and if severe may be a harbinger for a more gastrointestinal deposition. Patients are usually seen in clinic weekly or fortnightly for a month and then once every month. At the time of clinic visits complete blood count, serum tumour markers and liver function tests are assayed. Cross sectional imaging with CT/MRI is performed between 60-90 days following treatment to avoid radiation therapy tumour edema as erroneously being interpreted as progression. These decrease attenuation changes in the hepatic parenchyma may be noted on CT and are largely reversible [16]. 18F-fluorodeoxyglucose Positron Emission Tomography (PET) scans may be of use in cases of discordance where tumour markers are not elevated and CT scans suggest progression or to distinguish the site of progression in the presence of extra-hepatic disease when not evident by other standard means [17].

## CLINICAL APPLICATIONS

Early studies demonstrated the feasibility of Y90 microsphere therapy for a variety of disease types [18,19].Y90 microsphere therapy have since been applied principally for the treatment of unresectable hepatic metastatic colorectal and hepatocellular carcinoma.

One of the first systematic application towards the treatment of a specific tumour type was seen for colorectal cancer [20]. The application of Y90 resin microspheres to a patient population with hepatic colorectal metastases demonstrated favorable responses, augmented with the addition of hepatic arterial 5FU [21,22]. Encouraged by these results, a phase III trial in patients with laparotomy proven hepatic-only metastatic disease and resected primary was performed. In this pivotal trial, patients were randomised to receive intra-arterial FUDR with or without a single dose of the resin microspheres [23]. The results demonstrated a benefit in all clinical indices favoring the combination therapy, specifically a time to tumour progression of 15.9 versus 9.7 months (p<0.01) and formed the basis for FDA approval in the US. An important lesson was learnt during this trial; the majority of patients developed extra-hepatic disease that adversely affected survival and this observation was supported by a separate large clinical experience [24]. Furthermore, with the introduction of irinotecan and oxaliplatin with systemic 5 FU, liver directed therapy with intra-arterial FUDR was no longer the standard of care at the time of trial completion.

In order to address these shortcomings, a phase II randomised trial and two phase I trials that combined systemic 5 FU/LV and the 5 FU based regimens of oxaliplatin and irinotecan respectively, were performed with enrollment in Australia and Europe. In the randomised phase II trial, responses were significantly augmented with the addition of the Y90 microspheres (8 PR versus 0 PR) [25]. The phase I trials unequivocally demonstrated that these systemic therapies could be safely combined. Furthermore such combinations were within the dose ranges administered in clinical practice and generated robust responses (PR + CR of 90% with FOLFOX-4 regimen) [26, 27].

**Figure 7:**
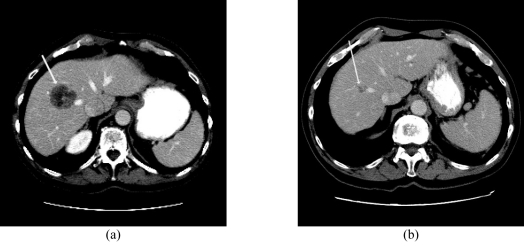
Partial response to Y90 microsphere therapy with the use of 1.61 GBq glass microspheres in an 83 yr old man with metastatic colorectal cancer; Contrast enhanced CT scan (a) before treatment demonstrates a low attenuation area in the right lobe of the liver (arrow) consistent with a metastasis with (b) significant reduction in size 12 months after treatment.

In the United States however, the application of this therapy has been relegated to treating patients who have failed multiple chemotherapy regimens most often as a single agent and then without a fixed combinatorial chemotherapy regimen (Figure 7). Responses in this highly pre-treated population have been demonstrated, but as expected, are significantly lower than the chemo-naïve population [15].

## HEPATOCELLULAR CARCINOMA

The other major therapeutic area that has been used with Y90 microsphere therapy has been in the treatment of unresectable hepatocellular carcinoma (HCC). Extensive experience has been gained in the treatment of HCC with resin microspheres in Asia and with glass spheres in the United States.

In Asia, Y90 resin microspheres as an effective treatment option was first delineated by an 18 patient phase I/II trial and supported by a observational study in 71 patients conducted by the same group. These studies found that tumour response and clinical benefit was proportional to the dose delivered; patients receiving >120 Gray survived 55.9 weeks compared with 26.2 weeks for those patients that received <120 Gray. Repeat treatments with Y90 microspheres provided additional survival benefits [28,29]. A Canadian study published in 2000 reported on 22 patients to determine response parameters, survival and toxicity after intra-arterial injection of ^90^Y glass microspheres [30]. 20 were evaluated for efficacy including 9 patients who were Okuda stage I and II, and 11 patients who were Okuda stage III. The median dose delivered was 104 Gy (range 45-145 Gy). Interestingly the median survival of 54 weeks (range 7-180 weeks) and the trend for enhanced survival with higher doses (> 104 Gy) was similar to the results seen for resin microspheres.

**Figure 8:**
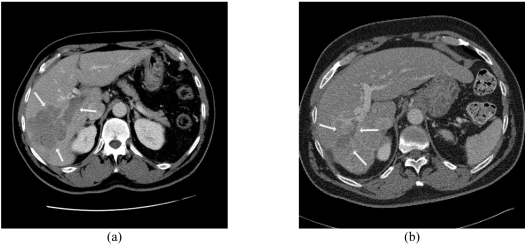
Portal vein invasion from right lobe hepatocellular carcinoma; (a) before and (b) after treatment with 2.39 GBq glass microspheres in a 72 yr old man. A partial response with regression of the tumour thrombus (arrows) is noted. Complete normalisation of tumour marker (AFP) was obtained.

Several retrospective patient studies have emerged from the centers treating with glass Y90 microspheres in the USA. In an analysis by Carr et al. in 65 patients, 38% had partial responses while the median survival duration for Okuda Stage I and II patients was 649 and 302 days respectively [31]. Geschwind reported on 80 patients from a relatively large database of 121 patients who were treated with glass microspheres [32]. Patients were staged using the Child-Pugh, Okuda, or Cancer of the Liver Italian Program (CLIP) scoring systems. Survival was found to be 628 and 324 days for Okuda I (68%) and II (32%) patients respectively. Data from an in-depth subset analysis in 121 patients elucidated factors that predicted high 3-month mortality. These included infiltrative tumour, liver replacement by tumour ≥ 70%, elevation in liver enzymes (ALT/AST) ≥ 5 x ULN, a combination of tumour volume ≥ 50% and albumin < 3 g/dL, bilirubin elevation ≥ 2 mg/dL [33]. Y90 microsphere treatment has resulted in the downstaging of non-resectable disease to either be treatable by transplantation, resection or RFA [34] or transplant [35] (Figure 8).

In summary, the tumours treated with Y90 microspheres have responded to therapy. This is evidenced by reduction in tumour volume and markers, ability to convert to a resectable status, and improvements in the time to tumour progression. However, there are many unanswered critical questions; who would be the ‘optimal’ patient? Should the radiation dose be fractionated, and at what dose and frequency? Does a dose response to tumour volume correlation exist? Can other newly developed systemic therapies be integrated safely? If so, what sequence should the therapy be initiated? Randomised clinical trials and registry data will assist answering these important questions.

## TOXICITIES

The incidence of complications is low if patient selection is appropriate and delivery technique is meticulous. Post treatment fatigue occurs uniformly with varying degrees of severity and is almost always transient lasting 10-14 days. Abdominal pain, nausea and vomiting are common but manageable via conservative means. Severe symptoms are relatively uncommon and should alert the clinician about possible extrahepatic microsphere deposition and consequences thereof.

Pancytopenia that had been reported in the earliest version of the Y90 microsphere has not been reported with the newer agents that are in current clinical use [19]. Radiation pneumonitis following lung exposure can occur when the dose to the lung exceeds 30Gy [12]. As a testament to the validity of utilising Tc99m MAA scan to calculate potential lung exposures, in over 3000 doses administered in the US, no cases of this complication have been reported. Radiation gastritis and gastrointestinal ulceration occur in less than 10% of cases; the vast majority of such cases have been managed conservatively without sequelae [13]. Gall bladder wall edema is a common finding following treatment, but cholecystitis requiring a cholecystectomy is rare [36]. Radiation induced liver disease (RILD), erroneously called radiation hepatitis, a *form fruste* of hepatic veno-occlusive disease. This presents clinically as a triad of hepatomegaly and anicteric ascites. Steroids have been the mainstay of therapy and have a poor and variable success at altering the natural history of the disease process and in most instances hepatic insufficiency associated morbidity ensues. Fortunately the reported incidence of RILD is low. Translation of dosimetry research may eventually mitigate the incidence of this entity.

## CONCLUSION

Y90 microspheres represent an intriguing therapy for the treatment of liver cancer. However, the utility of Y90 microsphere therapy remains to be determined within the context of the other currently available therapies. Standardisation of dosimetry and treatment techniques, achievable only in the robust randomised clinical trials, are necessary to arrive at conclusions that support clinical effectiveness. Registry data will be necessary to provide guidance on therapeutic effectiveness and for disease types for which clinical trials are not historically feasible due to their low incidence and for many patients who do not meet traditional eligibility criteria. Such efforts are underway.
